# “Omics” in traumatic brain injury: novel approaches to a complex disease

**DOI:** 10.1007/s00701-021-04928-7

**Published:** 2021-07-17

**Authors:** Sami Abu Hamdeh, Olli Tenovuo, Wilco Peul, Niklas Marklund

**Affiliations:** 1 Department of Neuroscience, Neurosurgery, Uppsala University, Uppsala, Sweden; 2 Turku Brain Injury Centre, University of Turku and Turku University Hospital, Turku, Finland; 3 Leiden University Neurosurgical Center Holland, HMC, HAGA & LUMC, The Hague & Leiden, Netherlands; 4 Department of Clinical Sciences Lund, Neurosurgery, Lund University, Skåne University Hospital, Lund, Sweden

**Keywords:** Traumatic brain injury, Mechanisms, Epigenetics, Metabolomics, Genetics

## Abstract

**Background:**

To date, there is neither any pharmacological treatment with efficacy in traumatic brain injury (TBI) nor any method to halt the disease progress. This is due to an incomplete understanding of the vast complexity of the biological cascades and failure to appreciate the diversity of secondary injury mechanisms in TBI. In recent years, techniques for high-throughput characterization and quantification of biological molecules that include genomics, proteomics, and metabolomics have evolved and referred to as omics.

**Methods:**

In this narrative review, we highlight how omics technology can be applied to potentiate diagnostics and prognostication as well as to advance our understanding of injury mechanisms in TBI.

**Results:**

The omics platforms provide possibilities to study function, dynamics, and alterations of molecular pathways of normal and TBI disease states. Through advanced bioinformatics, large datasets of molecular information from small biological samples can be analyzed in detail and provide valuable knowledge of pathophysiological mechanisms, to include in prognostic modeling when connected to clinically relevant data. In such a complex disease as TBI, omics enables broad categories of studies from gene compositions associated with susceptibility to secondary injury or poor outcome, to potential alterations in metabolites following TBI.

**Conclusion:**

The field of omics in TBI research is rapidly evolving. The recent data and novel methods reviewed herein may form the basis for improved precision medicine approaches, development of pharmacological approaches, and individualization of therapeutic efforts by implementing mathematical “big data” predictive modeling in the near future.

**Supplementary Information:**

The online version contains supplementary material available at 10.1007/s00701-021-04928-7.

## Introduction

Traumatic brain injury (TBI) is a leading cause of mortality and morbidity. In Europe, 262 per 100,000 inhabitants are annually hospitalized for TBI, and in the USA, TBI accounts for one-third of all injury-associated deaths [20, 92]. It affects patients of all ages in developed and developing countries alike. Survivors are frequently left with debilitating deficits in motor, sensory, cognitive, and emotional functions with marked impact on their quality of life [76, 78, 79, 103]. In children and young to middle-aged adults, TBI is the most important cause of death and disability, and thus, it has profound socioeconomic impact [64]. TBI is most frequently caused by motor vehicle accidents in young and middle-aged adults, while in the pediatric and the elderly population, falls account for the majority [76, 92, 114]. The consequences of TBI persist long after the initial trauma and are not always immediately recognized [79].

Importantly, TBI is a disease process, initiated at the trauma event, and then aggravated by a complex series of secondary insults and injury cascades that progress over days, months, to years [79]. The initial, primary injury marks the beginning of a series of pathological events in neuronal cells including calcium influx, mitochondrial damage, and increase in free radicals causing disturbances in energy metabolism, extensive damage to the cytoskeleton, and both necrotic and apoptotic cell death [71, 126]. In addition, progressive neuropathology is frequently observed well into the chronic phase of the disease with persistent neuroinflammation, white matter degeneration, and progressive brain atrophy at long-term [16, 57]. Plausibly, this contributes to the established risk increase for early-onset Alzheimer’s disease (AD), tauopathies, and other neurodegenerative disorders observed in TBI survivors [21, 37, 40, 58, 79, 95].

The list of clinical and experimental publications evaluating pharmacological strategies for the modulation of the secondary injury cascades is exhaustive. Unfortunately, to date, there is still no pharmacological drug with proven efficacy for human TBI [11, 115]. Instead, progress in TBI treatment and outcome has been achieved mainly by improved prehospital management, rapid and targeted surgical intervention, and implementation of focused neurointensive care monitoring and treating avoidable secondary insults such as hypoxia, hypotension, hypo-/hyperglycemia, pyrexia, epileptic seizures, and increased intracranial pressure [31]. Initially, early improvements led to a decreased mortality after severe TBI in the last decades of the twentieth century [31]. Nonetheless, since then, TBI outcomes have been largely constant [69], mainly owing to the limited knowledge of the underlying molecular pathophysiology.

One important reason for the failure of trials is the heterogeneity of TBI [59]. The currently used TBI classifications remain inadequate in appreciating the heterogeneity of TBI and its differences in the pathophysiology of secondary brain damage. Frequently, TBI is classified by either pathoanatomical terms such as focal or diffuse injury, or by its severity using the Glasgow Coma Scale (GCS) [104]. However, the heterogeneity of TBI remains a major barrier for the development of robust and reliable molecular biomarkers for diagnostic, monitoring, and prognostic purposes. Although many molecules have been proposed to reflect different aspects of TBI pathophysiology, an optimal set of biomarkers has not been developed [63]. In addition, research in TBI is hindered by the limited availability of reliable biological samples from patients, as samples from brain tissue, cerebrospinal fluid (CSF), and/or interstitial fluid (ISF) are difficult to obtain. Thus, there is an unmet need for advanced methods to facilitate research on TBI pathophysiology as well as development of reliable biomarkers and efficient pharmacological therapies.

## The field of omics

**Fig. 1:**
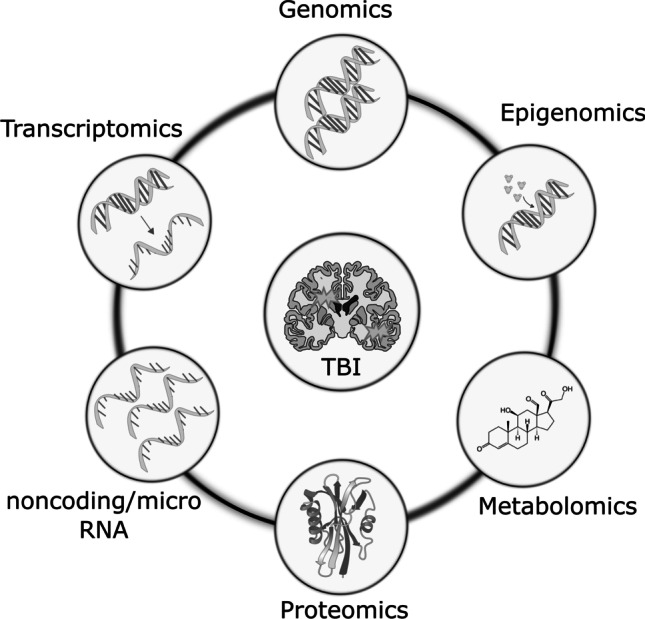
Omics refers to techniques for high-throughput characterization and quantification of biological molecules. These techniques provide possibilities to study function, dynamics, and alterations of molecular pathways in biological samples of normal cerebral and diseased states such as traumatic brain injury (TBI). Omics includes collective characterization and quantification of the organism’s genes (genomics), epigenetic mechanisms (epigenomics), genetic transcripts to RNA molecules (transcriptomics), proteins (proteomics), and metabolites (metabolomics)

**Fig. 2:**
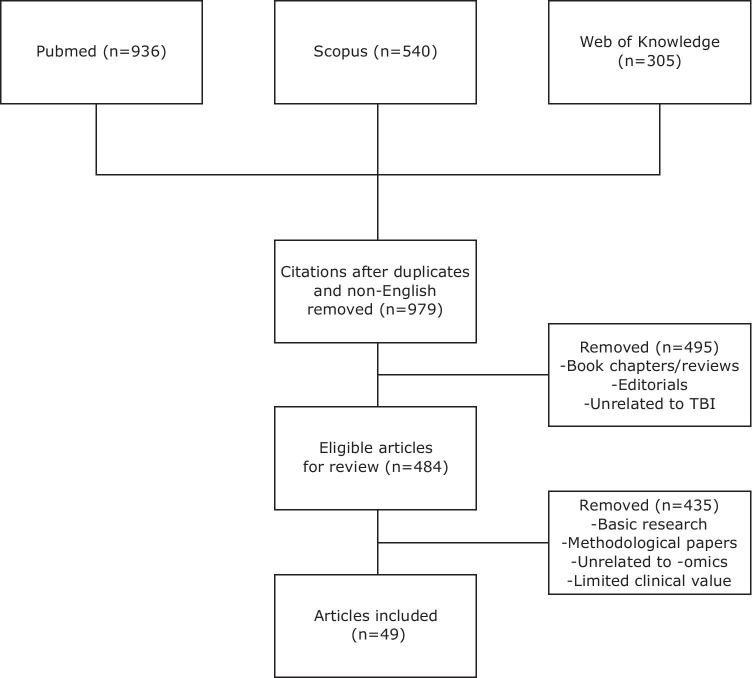
Flow diagram for search strategy for articles included in Supplementary Table 1

In recent years, techniques for high-throughput characterization and quantification of biological molecules have evolved. The study of genomics, epigenomics, transcriptomics, proteomics, and metabolomics is referred to as omics [61] (Fig. 1). These platforms provide possibilities to study function, dynamics, and alterations of molecular pathways in biological samples of normal and diseased states, including TBI [42]. Omics is a rapidly progressing multidisciplinary field, covering all aspects of the cell, tissue, and/or organism. Through advanced bioinformatics, large amounts of data from small biological samples can be analyzed in detail both qualitatively and quantitatively and provide valuable knowledge of pathophysiological mechanisms [107]. In addition, bioinformatic tools and statistical methods can aid in integrating data from various biological domains. By incorporating different aspects of TBI pathophysiology, omics may allow a more detailed understanding of broad cellular and molecular alterations [42]. Additionally, it can aid in the characterization of previously unknown neuropathophysiological processes and the discovery of diagnostic and prognostic biomarkers. In the near future, omics technology may form an integral part of precision medicine and individualized therapies for TBI [48]. This narrative review will focus on how omics can be applied in TBI to advance our understanding of the disease. A literature search was performed in PubMed, Scopus, and ISI Web of Knowledge for articles in English with the words “traumatic brain injury” together with one or a combination of the words “omics,” “multiomics,” “genomics,” “epigenomics,” “transcriptomics,” “proteomics,” and “metabolomics.” Articles were extracted and further screened (Fig. 2). Focus was on articles with clinical implications for TBI. The purpose of this review was to overview the high-throughput characterization and quantification of pools of biological molecules, characteristic for the omics field. For that reason, studies evaluating single genes or molecules were not included, other than when necessary for the relevance of the text. The discussion of the various modalities herein seeks to introduce omics techniques to illustrate their potential in TBI research and management.

## Genomics

The individual genetic composition affects the response, recovery rate, and outcome following TBI, and many genes are suggested to modify the progression and outcome. However, in contrast to genetics, which refers to the study of individual genes and their roles in disease, genomics aims to collectively characterize and quantify genes. Genomics is particularly applicable to disorders where a combination of genes and environmental factors are implicated, such as TBI. It involves high-throughput DNA sequencing and analysis of the function and structure of the complete genome by advanced bioinformatics.

In genome-wide association studies (GWAS), the entire genome is investigated for single-nucleotide polymorphisms (SNPs) that are statistically enriched compared with healthy controls. A large number of gene polymorphisms are evaluated (typically 0.5–2 million SNPs) which poses statistical challenges and requires adequate sample sizes of cases and controls [18]. Analysis of the entire genome can help the detection of previously unknown genes associated with a susceptibility to secondary injury mechanisms or poor recovery. Candidate SNPs have been successfully identified with GWAS in neurological disorders [8, 116]. To the best of our knowledge, no group has evaluated the genome globally using GWAS in TBI, although many studies evaluate SNPs in individual genes [reviewed in [137]]. Nonetheless, signature genes differentially expressed by TBI showed numerous overlaps between top GWAS hits in, e.g., AD, schizophrenia, and Parkinson's disease (PD) in a rodent model [81]. In a clinical study exploring variations in 18 SNPs in biomarker encoding genes, a S100B variant allele SNP was associated with improved long-term outcome post-TBI [91]. Furthermore, the entire mitochondrial genome was investigated for SNPs in patients with severe TBI [19]. Here, one SNP, A10398G, was associated with functional outcome at 6 and 12 months, while SNPs in T195, T4216, and A10398 were associated with the CSF lactate-to-pyruvate ratio in females only. Additionally, the mitochondrial DNA haplotype K was associated with favorable outcome in a large cohort of patients with TBI [12].

Further technological refinements termed “next-generation sequencing” methodologies permit high-throughput sequencing and identification of de novo variants with higher reliability [70, 89]. Methods include whole-exome sequencing (WES) and whole-genome sequencing (WGS), where nucleotides are determined in the exome or the genome, respectively. The exome constitutes ~ 2% of the entire genome and represents the gene coding sequence [93]. WES may detect variants related to protein structure and function, while WGS may be a more powerful tool for detecting disease-causing mutations in large-scale human genome studies [7]. This kind of approach is currently undertaken in the field of TBI by the international Genetic Associations in Neurotrauma (GAIN) consortium, which combines several well-characterized genetic biobanks from studies conducted during the last two decades.

## Epigenomics

**Fig. 3:**
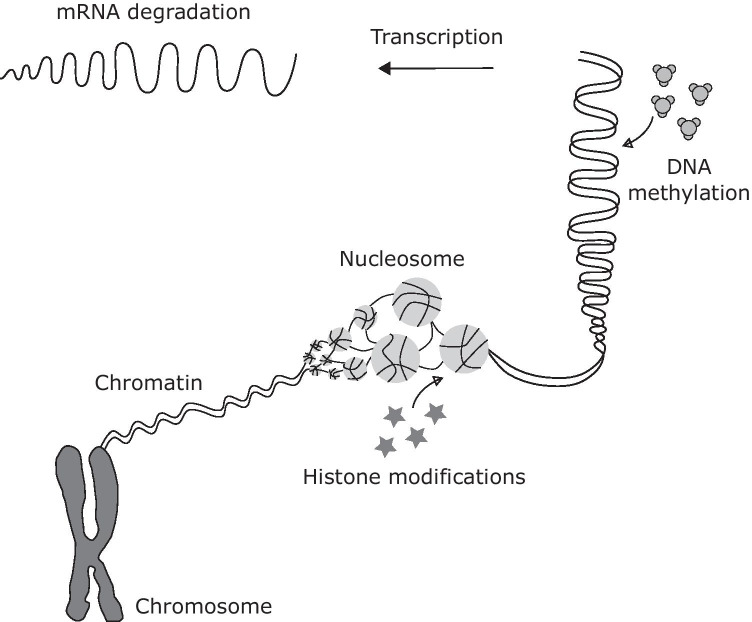
Epigenetic mechanisms may alter the expression of genes without corresponding changes in the DNA sequence. Epigenetics include DNA methylation or hydroxymethylation, post-translational histone modifications, changes in nucleosome positioning, and translational repression or through noncoding/microRNA. DNA is densely packed in the chromatin complex that form the chromosome, consisting of nucleosomes with DNA wrapped around histone proteins in a “bead on a string” formation. Post-translational histone modifications cause variability in chromatin packaging of DNA, allowing the DNA to be more or less readily available for transcription. Similarly, the arrangement of nucleosomes along the DNA sequence regulates gene expression by influencing the accessibility of DNA to the translational machinery. DNA methylation acts directly on the DNA sequence by adding methyl groups mainly on cysteine bases at cytosine–guanine-rich regions, while noncoding/microRNA regulate gene expression by either translational repression or mRNA degradation

Environmental factors may alter the expression of genes without corresponding changes in the DNA sequence through epigenetic modifications [121]. Epigenetic mechanisms include DNA methylation or hydroxymethylation, post-translational histone modifications, changes in nucleosome positioning. and translational repression or through noncoding/microRNA (miRNA, see separate paragraph, Fig. 3). They are involved in crucial cellular function during early development stages as well as later in life and are implicated in TBI [80]. These mechanisms may be rapid and dynamic or be stable and even heritable. There is compelling evidence of heritable epigenetic variations in plants, although relatively few examples in animals [43] and controversial in humans [43, 47]. In TBI, epigenetic mechanisms are still poorly understood although are implicated in the injury response to TBI, rate of recovery, and risk for future development of neurodegenerative disorders.

DNA methylation, catalyzed by DNA methyltransferase enzymes (DNMT), occurs mainly on cysteine bases at cytosine–guanine-rich regions of the DNA, CpG islands, and results in activation or repression of gene expression [9, 51, 125]. Epigenetic modifications are cell type– specific, and careful sample tissue selection is imperative. In the human brain, DNA methylation is involved in memory formation and storage [25, 83] and also modifies brain function after negative life impact experiences such as early age stress [39].

Following TBI, early global hypomethylation was observed in a rat TBI model [142]. In a focal TBI model in juvenile rats, DMNT-1 expression was upregulated in the prefrontal cortex and hippocampus [88]. Additionally, TBI resulted in large-scale DNA methylomic changes in the hippocampi in rodent TBI [81]. However, studies contradicting the role of DNA methylation in TBI exist. In one study using a system biology analysis, DNA methylation did not regulate chronic post-TBI transcriptomics changes following TBI in the rat [72].

Post-translational modification of histone proteins causes variability in chromatin packaging of DNA. DNA transcription is facilitated by loosely packed chromatin while inhibited by tightly packed chromatin. A variety of histone modifications exists, including acetylation, methylation, phosphorylation, and ubiquitylation, causing genes to be activated or repressed [51]. Histone acetylation can be increased using histone deacetylase (HDAC) inhibitors [36, 110], and HDAC inhibitors such as valproate and lithium decreased blood–brain barrier (BBB) permeability, reduced neural damage and inflammation, and improved cognitive and functional outcomes in experimental TBI [117, 130]. Thus, compounds modifying the epigenetic machinery show promise as a potential therapy following TBI.

In epigenome wide association studies (EWAS), the entire epigenome can be investigated for epigenetic modifications. These studies are possible using technology such as Illumina methylation assays or pyrosequencing. Such large observational studies may suffer from false-positive findings due to multiple testing or non-causal associations. However, they may also provide new insights into pathophysiology and develop novel biomarkers [10]. In a recent study, surgically resected human brain tissue from 17 patients with severe TBI was compared with brain biopsy samples from 19 patients with idiopathic normal pressure hydrocephalus. EWAS showed differential DNA methylation in 308 CpG sites in genes related to cellular/anatomical structure development, cell differentiation, and anatomical morphogenesis [2].

## Transcriptomics/miRNA

The transcriptome, estimated to be < 5% of the genome, represents the part of the genetic code that is transcribed into RNA molecules [34]. However, mechanisms resulting in variances of RNA molecules, such as alternative splicing, RNA editing, or alternative transcription initiation and termination sites, add complexity to transcriptional activity. Following TBI, a multitude of differentially expressed genes is found experimentally, identifying, e.g., inflammatory, cell signaling, and reduced pro-survival signatures [44, 52, 106, 127, 129, 139]. A study comparing an in vitro and an in vivo model of TBI demonstrated strong correlation in differentially expressed genes [66]. In a rat TBI study, RNA sequencing revealed 4964 regulated genes in the perilesional cortex and 1966 in the thalamus [74]. These high-throughput omics data can be used to evaluate novel candidate pharmacological therapies.

MiRNAs are short regulatory noncoding RNAs composed of 17–24 nucleotides. The miRNAs are not transcribed to protein; instead, they regulate gene expression by either translational repression or mRNA degradation [51]. In the brain, miRNAs play roles in synapse formation, neuronal network signaling, neuronal repair, and cell survival pathways [108, 128]. MiRNAs may be analyzed from either brain tissue, CSF, or blood, using sequencing or microarray methodologies. MiRNAs are abundant and stable and are expressed early following TBI, which make them attractive as biomarkers [28], and are found to discriminate TBI severities as well as patient with TBI from uninjured controls [98, 134]. MiRNAs have the advantage of being readily available in plasma samples, facilitating their use as biomarkers. Among the most promising miRNAs in TBI are miR-21, miR-16, and let-7i. MiR-21 is highly expressed after TBI and found to reduce brain edema in rodents [38]. Additionally, miR-21 was elevated in serum at days 1 and 15 in severe TBI but not in patients with mild TBI [27]. MiR-16, involved in apoptosis and cell cycle mechanisms, is increased in plasma in mild TBI and decreased in severe TBI [101]. The let-7 family, highly enriched in brain tissue, was upregulated both in serum and CSF after blast-induced TBI and is involved in the regulatory pathways of several neuroinflammatory cytokines [5, 105]. In addition, several studies have shown potential of miRNA panels for diagnostic and prognostic purposes in various biofluids [27, 46, 62, 86, 105, 134]. Although still early in their development, miRNAs are promising as potential clinical biomarkers for diagnostics, injury progression monitoring, and possible targets for individual precision medicine treatment.

## Proteomics

The protein composition of an organism is highly dynamic and can alter significantly in response to external stimuli. Analysis of the proteome, i.e., the total set of proteins produced by an organism, can reveal alterations in a multitude of biological processes following TBI [100]. The principal technique used in high-throughput proteomics is mass spectrometry (MS), since it is highly sensitive and specific, can identify proteins in small biological samples, and can identify a large number of different proteins [100]. An important limitation in MS-based proteomic research is the bias toward highly abundant proteins. There are numerous available techniques to deplete these highly abundant proteins and enable identification of important proteins with low abundance.[94]. Affinity-based proteomic methods may also be automated for efficient multiplexing of proteins at high-throughput, are flexible and highly sensitive, however require high-quality affinity reagents to provide reliable measurements [113, 132].

Proteins detected with proteomics reflect not only the underlying pathophysiological process, but also the proteome of the biological sample analyzed. Therefore, there is significant variability in protein expression between different brain regions [109].

The number of studies using proteomics technology after TBI is expanding in the evaluation of biomarkers, although their clinical use has not been established.

In surgically evacuated brain tissue from the frontal or temporal area in severe TBI, > 4000 proteins were identified of which 160 were overexpressed and five were downregulated compared to *postmortem* controls [133]. The altered proteins were involved in a multitude of biological processes, including glial cell differentiation and complement activation. Also, insoluble proteins such as those found aggregated in AD and chronic traumatic encephalopathy (CTE) can be characterized globally, providing a footprint of the total amount of insoluble proteins found in the analyzed sample. Recently, *postmortem* CTE prefrontal cortex was compared to controls, and > 700 proteins were increased or decreased in CTE, of which multiple were unique for CTE [15].

Analysis of structurally uninjured cortex from patients with severe TBI, sampled simultaneously with the insertion of an ICP monitor, revealed distinct alterations in protein expression between focal and diffuse TBI. The analysis of small brain tissue biopsies identified 51 up- or downregulated proteins in patients with diffuse axonal injury. Among these alterations, tau was increased, while proteins related to the antioxidant defense such as glutathione S-transferase were decreased [1].

In TBI, there are several brain-enriched protein biomarkers such as S100B, tau, neurofilament light (NF-L), and glial fibrillary acid protein (GFAP), among others. However, single biomarkers alone are not likely to reflect the multifaceted pathophysiology of TBI. One advantage of high-throughput proteomics is that candidate biomarkers can be measured in combination to increase diagnostic and prognostic accuracy. Recently, candidate blood biomarkers of TBI were tested together by affinity-based methods [49]. Blood samples from the TRACK-TBI databank were used in a protein array, where 21 of 72 proteins were identified as potential biomarkers using a multivariate analysis. Together, these proteins that were all related to the inflammatory response showed increased inflammatory signatures with positive CT findings and poor recovery. This approach, however, did not reach the sensitivity and specificity obtained with brain-enriched biomarkers. Alternatively, panels of brain-enriched and inflammatory biomarkers can be used in conjunction to improve prognostic accuracy [118]. A combination of protein biomarkers discovered by proteomics could be integrated with clinical and radiological biomarkers for better prognostication and surveillance of injury progression [136].

## Metabolomics

Metabolomics is the study of structures, functions, and interactions of metabolites in cells, tissues, and body fluids. The metabolome is the phenotypic expression of the genome and proteome and comprises groups of metabolites produced by the cell, tissue, organism, or any other respective entity. Every second, numerous metabolic processes take place in any living organism, producing measurable small molecules in various tissues and body fluids. Due to recent technical advances, the extent of the human metabolome has been realized, and this field is rapidly expanding.

The brain can be regarded as the most active organ in humans, utilizing one-fifth of the total energy and blood volume. Central nervous tissue has several metabolic pathways that are fairly specific for the CNS [41]. Thus, measuring the metabolic fingerprint of the brain and monitoring the temporal changes occurring in this fingerprint have the potential to produce very accurate and comprehensive data about the state of the brain [123]. Moreover, compared to proteomic profiling, metabolomic analysis is much less dependent on the BBB, because the measured molecules are smaller and thus more readily penetrate an intact BBB. Yet, metabolites are not fully independent of the BBB. Although some metabolites may diffuse freely through the BBB, other polar metabolites may have active transfer depending on their polarity, or their diffusion may depend on whether they are hydro- or lipophilic.

Metabolic analysis of the brain is not a fully novel approach, and clinical applications have in fact been in use for a while. These include magnetic resonance spectroscopy (MRS) and microdialysis of the brain interstitial fluid [13, 144]. Both of these focus on a very limited number of metabolites, which however are able to provide clinically useful information about the state of the brain, especially its energy metabolism. Both methodologies also have spatial limitations: MRS analysis must be focused in a certain brain area, and brain microdialysis measures the metabolic state mainly in the close proximity of the probe. Yet, microdialysis is able to give essential information to guide clinical care in severe brain insults, not available otherwise [138].

Metabolomic analysis can be performed in any fluid in the body, but here we concentrate on blood-based metabolomics since analysis of the CSF is rarely clinically feasible, and metabolomic profiling from other body fluids (saliva, urine, lacrimal fluid, etc.) has not been thoroughly evaluated in brain disorders. However, there is no reason why these other body fluids could not provide equally important information regarding the brain. Serum metabolome consists mainly of lipids and small polar metabolites (carbohydrates, amino acids, alcohols, polyols, organic acids, free fatty acids). Analysis of lipid metabolites is often called lipidomics [124]. Metabolites from any fluid can be analyzed using liquid or gas chromatography and MS from a very small volume. For known metabolites or panels of metabolites, simple and rapid detection can be done using small mass spectrometers, as most have experienced at airports.

It is self-evident that serum metabolome does not contain only brain-related metabolites but metabolites from all parts of the body. One of the major challenges in metabolomics is to determine the biological metabolic processes that produce the detected/measured metabolites. Using data libraries and maps of metabolic pathways, this is often possible. If the source of the metabolite can be determined, the challenge of anatomical location remains since metabolic processes are often largely similar in various cells in various organs. More simply, it is often impossible to determine if a metabolite is brain-related or derived from processes elsewhere in the body. This may, however, be a less important problem than assumed, since in TBI, the brain is not separated or independent from the rest of the body. Thus, systemic reactions to brain insults may well be equally important both diagnostically, therapeutically, and prognostically [102]. Metabolomics may potentially enable fairly accurate anatomic localization in the brain, as shown in experimental animals having brain region–specific signatures and responses to injury [53]. Metabolites have been also capable of differentiating gray matter from white matter injury in piglets [4].

TBI is man’s most complex disease and is associated with a highly complex and dynamic metabolic disruption. One of its main components is the energy crisis and energy failure, caused by, e.g., ischemia, hypoxia, mitochondrial failure, or increased energy need [17, 65, 82, 120]. The brain is enriched with different lipids due in part to the complex myelin structures, why lipidomic analysis may be especially useful when analyzing brain disorders. Currently, the research of metabolomics in TBI is still in its infancy. Circulating amino acids have differentiated severe TBI from milder cases [55] and been able to predict elevated ICP [56]. In a pioneer study, human serum metabolites were shown to associate strongly both with the severity and outcome of TBIs of all severities [90]. Several metabolites have been shown to be either up- or downregulated in human severe TBI [96]. A metabolite panel has also been able to separate patients with acute mild TBI from controls [32], and metabolites have been associated with both CT and MRI findings in TBI [29, 119].

To conclude, metabolomics holds great promise as a tool for diagnosis, monitoring, and prognostication of TBIs of all severities. They probably react more quickly upon pathophysiological changes when compared to proteins and enable point-of-care diagnostics. Since they also react rapidly to, e.g., altered medications, diet, and exercise, substantial bioinformatic work-up is needed to establish the best metabolite panels for different types of injuries and their temporal profile after TBI.

## Statistical challenges, artificial intelligence, and machine/deep learning

High-throughput omics technology provides a possibility to generate large amounts of data from biological samples. Much of this rapidly progressive knowledge has been stored for access in large and publicly distributed databases [68, 77]. The amount of generated data creates many opportunities for better understanding of TBI but also require application of robust statistical predictive modeling methods. In addition, the exponentially rising amounts of medical data produced from clinical research demand firm data storage solutions to guarantee security and patient integrity. For individualized precision medicine, data from different omics sources (i.e., multiomics) should be integrated and combined with clinical information. To date, omics research in TBI is still in its infancy, and most studies approach different aspects of TBI pathophysiology, leaving little space for consolidation of data from multiple sources. Additionally, the statistical modeling of TBI poses a challenge in view of the heterogeneity of the disease and since data generated by high-throughput technology may be measured in thousands to millions per sample [22]. This high dimensionality carries statistical difficulties such as sparsity, multicollinearity, model complexity, and model overfitting [112]. Multivariate statistical approaches to omics data, such as modified versions of partial least squares regression (PLS) and canonical correlation analysis (CCA), are required. These models should incorporate multiple biomarkers in multiple disease phenotypes. A system biology approach in multiomics, advocating integration and analysis of different biological processes in the organism simultaneously, poses demands on the performance of statistical models. Omics domains are not distinct and separable biological systems but rather represent different biomolecular data sources measuring the expression of various biological processes [122]. Therefore, no single omics modality can completely reflect the complexity of TBI, and a system biology approach is needed. To accomplish this, multiset techniques based on PLS and CCA are available [22]. In addition, network and enrichment analysis is valuable to identify molecules of pathophysiological significance and to understand the downstream flow of information from DNA to physiology [60]. However, there is no consensus for modeling, comparing, or benchmarking the performance of the various data analysis strategies. The latter is crucial as optimistic scientific opportunism increases the risk of inference on possibly “wrong” prognostic assumptions by coincidental statistical significance, caused by the existing ample amount of omics variables. Method selection is instead based on knowledge about the structure of the data and the research questions of interest. In the case of TBI, many studies suffer from low sample size albeit high dimensionality. Dataset integration is a potential mean to increase sample size, although often not feasible due to systematic variability in technology, protocols, and experimental conditions between studies [112].

Artificial intelligence and deep learning applications have huge potential in analyzing information from TBI datasets, as they may achieve higher accuracy and speed in data analysis. In deep learning, a subfield of machine learning, a layered structure of algorithms — an artificial neural network — is used to learn the application to draw inference from the data [84]. The recent progress is tremendous for image recognition and histopathological analysis [50, 143]. In TBI, deep learning applications exist for the detection of intracranial pathology from CT scans, performing in agreement with expert assessments [54], as well as for the detection of cerebral microbleeds on MRI [75]. Additionally, machine learning applications incorporating clinical information have been used for stratification of TBI phenotypes and prognosis [33, 99]. Deep learning applied to omics data has gained great interest, and the number of publications is increasing [141]. The challenges when applying deep learning to omics research relate to the data volume and quality needed to train the systems. Deep learning applications require large amounts of data for training which may not be available, and the quality of the learning depends on the quality of the input data. In addition, they may provide the desired prediction by using the input data but do not explain how the prediction was reached, i.e., the “black box” problem [67, 140, 141]. Nonetheless, artificial intelligence and deep learning may prove useful for omics research to provide clinically valuable conclusions as the amount and dimensionality of the data are expanding.

## Clinical applications and treatment possibilities

The study of the different biological domains of the organism in depth by omics technology is a concept of relevance for TBI. The implementation of omics in the clinic to aid decision-making, and to enable highly individualized medicine, has begun. Genome sequencing is in use to diagnose rare disorders [131], and multiomics approaches are developed to build predictive models of disease in the healthy individual [3, 14]. However, there are challenges to overcome before omics technology can become an integral part of clinical TBI practice. Although genomics has successfully been used to dissect genetic diseases [60], the pathophysiology of TBI is far more complex. Experimental studies of omics in TBI thus far use mainly lissencephalic animal models. Relevant animal models are essential for TBI research to enable exploration of pathophysiology and biomarkers. Nevertheless, the brains of humans and those of the lissencephalic rodents are vastly different and form a major barrier for successful translation of experimental research, particularly in the omics field in view of the tissue and cell specificity of the molecular alterations. To date, omics studies in TBI have not generated diagnostically or prognostically useful biomarkers to the clinics. Nonetheless, protein panels including S100B, neuron-specific enolase (NSE), ubiquitin C-terminal hydrolase L1 (UCH-L1), GFAP, and NF-L [97] have been tested in TBI for classification into severity type or prognostic purposes. Similarly, panels of metabolites in biofluids are being developed for diagnostic purposes [29]. The integration of omics and clinical data could further augment the diagnostic and/or prognostic accuracy [45]. Nevertheless, translation of preclinical biomarkers to the clinical setting has been hampered by lack of homogenization of target cohorts, inconsistency of study design, and reporting as well as lack of standardization of techniques for sampling and analysis of biological specimens [87]. Consequently, it is essential that future studies streamline study design, methodology, and reporting to allow reproducibility and pooling of data for effective translation into meaningful clinical use.

To date, omics analysis is still too expensive and laborious for bedside use. Nonetheless, technologies for fast, easy-to-use analytical devices able to provide on-site testing of different molecules are emerging and could prove valuable for TBI in the future [30]. Such devices, capable of high-throughput point-of-care analysis of DNA, RNA, proteins, and metabolites, would be particularly beneficial in resource-limited settings. High-throughput omics provides the advantage of integration of various biological domains to potentially augment diagnostic and prognostic specificity. However, in certain clinical situations, less strenuous single marker solutions may be more cost-efficient and adequate for the endpoint of interest [23].

In addition to the potential of omics to allow discovery of novel biomarkers or for the monitoring of secondary injury development, omics research may also generate candidate pharmacological compound. HDAC inhibitors, such as valproic acid and lithium, acting via histone modification, and overexpression of the miRNA let-7c-5p, have proven efficacy in rodent animal models [24, 110, 117, 135]. Non-hypothesis-driven in silico (computer stimulations) network approaches to drug discovery, using omics data to generate candidate pharmacological therapies, are promising in AD, PD, and epilepsy [6, 26, 35, 85, 111]. In TBI, this strategy has been tested using transcriptomics data [73], albeit without efficacy in an *in vivo* TBI model. Still, with further refinements, there is potential for further steps toward new therapeutic strategies.

## Conclusions

Traumatic brain injury is a devastating disease affecting millions worldwide, and there is a lack of effective therapies. Numerous studies evaluating pharmacological compounds in the treatment of TBI have failed, plausibly due to an incomplete knowledge of the underlying pathophysiology and disease heterogeneity. The rapidly evolving field of high-throughput omics technology such as genomics, epigenomics, transcriptomics, proteomics, and metabolomics enables detailed “big data” analysis of differential alterations in the molecular domains affected by TBI. Research on omics in the TBI context is merely emerging, and studies thus far are small, heterogenous, and do not allow generalized conclusions. Nonetheless, the development is rapid, and the potential is vast. Omics may provide opportunities for diagnosis, monitoring, and prognosis in TBI, as well as aid in the search for novel biomarkers and pharmacological therapies, with caution that prognostic modeling needs to be guided carefully by independent statisticians and outcome epidemiologists. Continuously increasing studies providing new data from larger cohort will pave the way for the use of omics as an integral part of an individualized approach to the TBI patient.

## Supplementary Information

Below is the link to the electronic supplementary material.

Supplementary file1 (DOCX 120 KB)
