# Supplementary material for: “Omics” in traumatic brain injury: novel approaches to a complex disease
Source: Acta Neurochir (Wien). 2021 Jul 17;163(9):2581–94. doi: 10.1007/s00701-021-04928-7 (PMC8357753; doi:10.1007/s00701-021-04928-7)
Supplement: Supplementary file 1 — Supplementary file1 (DOCX 120 KB) [file 701_2021_4928_MOESM1_ESM.docx]

Supplementary table 1: Relevant publications of -omics in traumatic brain injury

| Reference | Type of injury | Numbers | Time post-injury | Biological sample | Technique | Key findings |
| --- | --- | --- | --- | --- | --- | --- |
| *Genomics* |  |  |  |  |  |  |
| Meng et al (transcriptomics) [1] | FPI rats | TBI n=10,  sham n=10 | 24h and 7 days | Brain, blood | RNA-seq, qPCR, RRBS system biology approach | 268 and 1215 unique genes in hippocampus and leukocytes, large scale DNA methylomic changes, relationship between DNA methylation and expression, genes differentially expressed by TBI overlaps with top GWAS in AD, schizophrenia, and PD among others |
| Osier et al [2] | sTBI | TBI n=305 (Caucasians) | Not reported | CSF, blood | Multiplex assay platform of 18 biomarker encoding genes | S100B variant allele SNP (rs1051169) associated with higher GOS scores 3-24 m post TBI |
| Conley et al [3] | sTBI | TBI n=136 (Whites) | Not reported | CSF, blood | Mitochondrial sequencing microarray, qPCR | SNP A10398G associated to functional outcome at 6 and 12 m, SNPs in T195, T4216 and A10398 associated with CSF lactate:pyruvate ratio in females |
| Bulstrode et al [4] | Moderate- and sTBI | TBI n=1094 | Acute period | Buccal swab, blood | PCR, RFLP, mitochondrial DNA | Mitochondrial DNA haplotype K associated with favorable outcome |
| *Epigenomics* |  |  |  |  |  |  |
| Zhang et al [5] | Weight drop TBI rats | TBI n=46,  controls n=5 | 6-96h post-TBI | Brain | IHC | Global hypomethylation 1-2 days post- injury, sub-population of reactive microglia/microphages the major source |
| Mychasiuk et al [6] | Weight drop TBI juvenile rats | TBI n=7,  sham n=7 | 16 days post-TBI | Brain | qRT-PCR | DMNT-1 expression was upregulated in the prefrontal cortex and hippocampus. relationship between diet and specific genetic and epigenetic changes |
| Haghighi et al [7] | Shock tube overpressure injury rats | TBI n=4  sham n=4 | 8m post-TBI | Brain | ERRBS | DNA methylation perturbations in 458 and 379 genes in neurons and glia, respectively. Enrichment in neuronal genes involved in cell death and survival, nervous system development and function |
| Wang et al [8] | CCI mice | TBI n=41, sham n=29 | 6h-3d post-TBI | Brain | qPCR | Differentially expressed hypomethylated and hypermethylated mRNA transcripts in TBI |
| Lipponen et al [9] | FPI rats | TBI n=14, sham n=11 | 3m post-TBI | Brain | MBD- and RNA-seq | Transcription factors Tp73, Cebpd, Pax6, and Spi1 regulate chronic transcriptomics changes post-TBI, DNA methylation not a major regulator |
| Gao et al [10] | CCI immature rats | TBI n=15,  sham n=15 | 6-72h post-TBI | Brain | IHC, WB | Hippocampal histone H3 acetylation and methylation decreased in CA3 |
| Shein et al [11] | Weight drop TBI mice | TBI n=19,  sham n=19 | 3-21d post-TBI | Brain | IHC, WB | HDAC inhibitor ITF2357 administered 24 h after injury improved neurobehavioral recovery, decreased neurodegeneration and reduced volume loss |
| Tai et al [12] | CCI rats | TBI+treatment n=8, TBI+vehicle n=8, sham n=8 | 3-7d post-TBI | Brain | IHC,WB | Valproate increased histone acetylation, decreased contusion volume increased motor function |
| Abu Hamdeh et al [13] | sTBI | TBI n=17,  iNPH n=19 | 4-240h post-TBI | Brain (evacuated contusions) | Ilumina methylation assay, pyroseq, IHC | Differential DNA methylation in APP, tau and NF genes |
| *Transcriptomics* |  |  |  |  |  |  |
| Israelsson et al [14] | CCI mice | n=3-9 animals/timepoint | 0h-3weeks post-TBI | Brain | qRT-PCR, gene microarray | Increased transcript levels characterizing reactive astrocytes, oligodendrocytes, and microglia. |
| Samal et al [15] | CCI rat | TBI n=6,  sham n=6 | 24h post-TBI | Brain | qRT-PCR, gene microarray | 193 upregulated, and 21 downregulated transcripts, primarily inflammatory cytokines |
| Zhang et al [16] | FPI rats | TBI n=39 | 30min-21d post-TBI | Brain | Gene microarray | Cell surface receptor-linked signaling, response to wounding and signaling pathways overrepresented |
| White et al [17] | CCI rats | NA | 24h post-TBI | Brain | IHC, gene microarray | IPA revealed top biological functions associated to TBI were related to inflammation, also on contralateral side |
| Von Gertten et al [18] | Weight drop TBI rats | TBI n=13,  sham n=2 | 1 and 4d post-TBI | Brain | Gene microarray | Genes with biological function clustered to immune response were upregulated 4 days, but not 1 day post-TBI |
| Hellmich et al [19] | FPI rats | TBI +treatment (30 min pre-TBI, metyrapone or carbenoxolone) n=12, TBI+saline n=6  Sham+saline n=6 | 24h post-TBI | Brain | Gene microarray | Metyrapone and carbenoxolon attenuated expression of genes in the apoptosis, death receptor, stress signaling and oxidative phosphorylation pathways |
| Lamprecht et al [20] | In-vitro hippocampal slice cultures, FPI immature rats, TBI post-mortem tissue | NA | 1-24h post-TBI | Brain | IHC,WB,gene microarray | Strong correlation in differentially expressed genes in in-vitro and in-vivo models. Gene expressing sorting protein related receptor with A-type repeats (SORLA), a protein with mutations linked to AD, down-regulated in TBI |
| Lipponen et al [21] | FPI rats | TBI n=5, sham n=5 | 3m post-TBI | Brain | RNA-seq | RNA sequencing revealed 4964 regulated genes in the perilesional cortex and 1966 in the thalamus. LINCS analysis identified Desmethylclomipramine as a potential modulating compound in TBI. |
| Lipponen et al [22] | Neuronal cultures, FPI rats | TBI n=26 (vehicle n=15, clomipramine n=11) | 28d post-TBI | Brain, blood | IHC, gene microarray, qPCR | Clomipramine failed to prove efficacy in TBI |
| *microRNA* |  |  |  |  |  |  |
| Qin et al [23] | Mild, moderate and sTBI | TBI n=90, controls n=30 | <24h post-TBI | Blood | miRNA microarray, qRT-PCR | Different expression profiles in plasma miRNAs among patients with mild to severe TBI. miR-3195 and miR-328-5p may distinguish mild and moderate TBI from sTBI |
| Yang et al [24] | Mild, moderate and sTBI | TBI n=76, controls n=38 | <24h and daily sampling for 21 d | Blood | qRT-PCR | miR‐93, miR‐191, and miR‐499 levels increased in TBI at all time points, , higher in sTBI and in patients with poor outcomes |
| Ge et al [25] | FPI rats | Total n=68 (TBI with up-and downregulated miR-21 and controls) | 3-14 days post-TBI | Brain | IHC, qRT-PCR | Upregulated miR-21 level conferred a better neurological outcome of TBI, and alleviated TBI-induced secondary BBB damage and loss of tight junction proteins |
| Harrison et al [26] | CCI mice | TBI n=15, sham n=15 | 7 days post-TBI | Brain (extracellular vesicles) | IHC, RNAseq, electron microscopy | Expression of miR-212 decreased, while miR-21, miR-146, miR-7a, and miR-7b were increased by TBI, miR-21 showed the largest change. |
| Di Pietro et al [27] | Mild and sTBI | Discovery: TBI n=10, controls n=5  Validation: TBI n=90, controls n=30 | 1 and 15 d post TBI | Blood | miRNA microarray, qPCR | miR-425-5p and miR-502) downregulated in mild TBI at early time-points and miR-21 and miR-335 upregulated in sTBI |
| Redell et al [28] | Mild and sTBI | TBI n=10 sTBI, n=11 mild TBI, n=16 with OI  control n=10 | <3d post-TBI | Blood | miRNA microarray, qRT-PCR | 52 microRNAs altered after sTBI, 33 decreased and 19 increased. miR-16, miR-92a, and miR-765 good markers of sTBI |
| Balakathiresan et al [29] | Shock tube TBI rats | TBI n=12, control n=6 | 3 and 24h post-TBI | Blood, CSF | miRNA microarray, qPCR | let-7i elevated in both serum and CSF after blast TBI |
| Sajja et al [30] | Shock tube TBI mice | TBI n=8, controls n=8 | 4d post-TBI | Blood | RNAseq | miR-127 increased in all groups let-7a, b, and g reduced in the 17 × 3 and 20 psi groups, and let 7d increased 17 psi group |
| Mitra et al [31] | Mild, moderate and sTBI | Mild TBI n=12, mild TBI+risk of PCS n=, moderate-sTBI n=7 | 1, 5 and 30d post-TBI | Blood | miRNA microarray, qPCR | mir142-3p and mir423-3p differentiated mild head injury into those at greater risk of amnesia and PCS. These miRNAs demonstrated a decrease over time |
| Hicks et al [32] | Pediatric mild and sTBI | sTBI n=7  mild TBI n=60, controls n=21 | sTBI 1, 4-7 and 8-17d post-TBI, mild TBI <14d post-TBI | CSF, saliva | RNAseq | Six miRNAs had parallel changes in both CSF and saliva (miR-182-5p, miR-221-3p, mir-26b-5p, miR-320c, miR-29c-3p, miR-30e-5p) |
| Ko et al [33] | Shock tube TBI mice, TBI (severity not specified) | TBI mice and sham (blinded set) n=77, TBI human and controls (blinded set) n=82 | TBI mice 1h-14d post-TBI, TBI human <120h post-TBI | Brain, blood (extracellular vesicles) | RNAseq, qPCR | miRNA signatures identified TBI patients from healthy controls (AUC = 0.9) |
| *Proteomics* |  |  |  |  |  |  |
| Cheng et al [34] | Cell culture, CCI rats, mild-moderate and sTBI | TBI rats n=80, sham n=80, TBI humans n=20 | 24h post-TBI | Brain, CSF | IHC, LC-MS/MS, WB, ELISA | Proteins associated with TTM were identified. Changes in plasminogen, antithrombin III, fibrinogen, gamma chain and transthyretin were verified using TBI rat brain tissues and TBI human CSF samples |
| Song et al [35] | Pendulum-striker, repetitive TBI rats | TBI n=18, sham n=9 | 1d, 7d and 6 months post-TBI | Brain | LC-MS/MS, WB | 237 proteins significantly changed in TBI, cAMP signaling, cell adhesion, autophagy, myelination, microtubule depolymerization and brain development were over-represented |
| Thelin et al [36] | CCI rats (hypoxia/normoxia) | TBI n=50, sham n=20, naïve n=3 | 1-28d post-TBI | Blood | Antibody-based microarray (Luminex) | Complement factor 9, complement factor B and aldolase c detected at higher levels 1d post-TBI. Hypoxia inducing factor 1α, amyloid precursor protein and WBSCR17 increased over weeks. S100A9 levels higher in hypoxic rats |
| Xu et al [37] | sTBI | TBI n=12, controls n=8 | ≤56 h | Brain (evacuated contusions) | LC-MS/MS | >4000 proteins identified, 160 overexpressed and 5 downregulated compared to *postmortem* controls. Altered proteins involved in multiple biological processes, including glial cell differentiation and complement activation |
| Cherry et al [38] | *Postmortem* CTE | CTE n= 45, AD n=23, controls n= 15 | Not applicable | Brain | IHC, LC-MS/MS | >700 proteins increased or decreased in CTE, of which multiple were unique for CTE. NADPH dehydrogenase quinone 1 (NQO1) showed significant enrichment in CTE and correlated with increasing CTE stage |
| Abu Hamdeh et al [39] | sTBI (focal/diffuse) | TBI n=16, iNPH n=11 | 4-175h post-TBI | Brain (cortical biopsy) | IHC, LC-MS/MS, WB | Differential protein expression between focal and diffuse TBI. 51 up- or down-regulated proteins in diffuse axonal injury. Among these, increased tau and decreased glutathione S-transferase |
| Huie et al [40] | Mild, moderate and sTBI | TBI mild n=104, TBI moderate n= 7, sTBI n=19 | <24h post-TBI | Blood | Antibody-based microarray | Proteins in microarray, all related to the inflammatory response, showed increased inflammatory signatures with positive CT findings and poor recovery |
| Posti et al [41] | Mild, moderate and sTBI | TBI n=160 | <24h post-hospital dmission | Blood | Antibody-based assay | Neurofilament light (NF-L), glial fibrillary acidic protein (GFAP), and tau were the best in discriminating CT-negative and CT-positive patients, both in patients with mTBI and with all severities |
| *Metabolomics* |  |  |  |  |  |  |
| Ritzel et al [42] | CCI mice | TBI n=25, sham n=25 | 1-14 and 60d post-TBI | Bone marrow, blood, thymus, and spleen | WB, ELISA |  |
| Baker et al [43] | CCI piglets | TBI n=16 | Pre-TBI, 24h and 7d post-TBI | Brain, blood | GC-MS | Differences in biochemical response between gray and white matter, white matter displaying greater metabolic change |
| Jeter et al [44] | Mild and sTBI | Mild TBI n=18, sTBI n=20, OI n=15, controls n=20 | <24h post-TBI | Blood | LC-MS/GC-MS | L-arginine, citrulline, ornithine, and hydroxyproline reduced and creatine increased in sTBI |
| Jeter et al [45] | Mild and sTBI | Mild TBI n=18, sTBI n=20, OI n=15, controls n=20 | <24h post-TBI | Blood | LC-MS/GC-MS | Combination of three branched-chain amino acids with changed levels post-TBI identified patients with sTBI and elevated intracranial pressure |
| Orešič et al [46] | Mild, moderate and sTBI | Discovery: TBI n=144, OI n=22  Validation: TBI n=67, OI n=27 | <12h post-hospital dmission | Blood, ISF | GC-MS | Two medium-chain fatty acids (decanoic and octanoic acids) and sugar derivatives including 2,3-bisphosphoglyceric acid are strongly associated with TBI severity. Addition of the metabolites to the CRASH model, improved prediction of outcomes |
| Fiandaca et al [47] | Mild TBI | TBI n=69, controls n=77 | 6h and 2-14d post-TBI | Blood | LC-MS | A 6-metabolite panel was identified and objectively classified mTBI from controls, at 6h 2, 3, and 7d post-TBI |
| Dickens et al [48] | Mild, moderate and sTBI | Discovery: TBI n=144, OI n=28  Validation: TBI n=188, OI n=27 | <12h post-hospital dmission | Blood, ISF | GC-MS | A combination of six metabolites (two amino acids, three sugar derivatives, and one ketoacid) could discriminate patients with intracranial abnormalities on CT and patients with a normal CT |
| Thomas et al [49] | Mild, moderate and sTBI | TBI n=96 | <12h post-hospital dmission | Blood | GC-MS | Two metabolic predictors (erythronic acid and *myo*-inositol), together with neurofilament light polypeptide (NF-L), discriminated positive and negative structural MRI findings |

N=numbers, FPI: Fluid percussion injury, TBI=traumatic brain injury, RNA-seq=RNA sequencing, qPCR=Quantitative real-time polymerase chain reaction, RRBS=reduced representation bisulfite sequencing, CSF=cerebrospinal fluid, m=months sTBI=severe traumatic brain injury, GOS=Glasgow outcome scale, PCR= polymerase chain reaction, RFLP= restriction fragment length polymorphism, IHC=immunohistochemistry, qRT-PCR=relative quantitative reverse transcription – polymerase chain reaction, DMNT-1=DNA methyltransferase enzyme-1, ERRBS=expanded reduced representation bisulfide sequencing, CCI=controlled cortical impact, MBD-seq=methyl-binding domain sequencing, WB=western blot, CA=cornu ammonis, HDAC=histone deacetylase, iNPH=idiopathic normal pressure hydrocephalus, APP=amyloid precursor protein, NF=neurofilaments, IPA= Ingenuity Pathway Analysis, LINCS=The Library of Integrated Network-Based Cellular Signatures, OI=orthopedic injuries, PSI=pounds per square inch, PCS=post-concussive syndrome, , LC-MS/MS= **Liquid Chromatography with tandem mass spectrometry, ELISA=**enzyme-linked immunosorbent assay, TTM= targeted temperature management, CTE=chronic traumatic encephalopathy, iNPH=idiopathic normal pressure hydrocephalus, GC-MS=Gas chromatography–mass spectrometry, ISF=interstitial fluid through microdialysis, CT=computed tomography

1. Meng, Q., et al., *Traumatic Brain Injury Induces Genome-Wide Transcriptomic, Methylomic, and Network Perturbations in Brain and Blood Predicting Neurological Disorders.* EBioMedicine, 2017. **16**: p. 184-194.

2. Osier, N.D., et al., *Variation in Candidate Traumatic Brain Injury Biomarker Genes Are Associated with Gross Neurological Outcomes after Severe Traumatic Brain Injury.* J Neurotrauma, 2018. **35**(22): p. 2684-2690.

3. Conley, Y.P., et al., *Mitochondrial polymorphisms impact outcomes after severe traumatic brain injury.* J Neurotrauma, 2014. **31**(1): p. 34-41.

4. Bulstrode, H., et al., *Mitochondrial DNA and traumatic brain injury.* Ann Neurol, 2014. **75**(2): p. 186-95.

5. Zhang, Z.Y., et al., *Global hypomethylation defines a sub-population of reactive microglia/macrophages in experimental traumatic brain injury.* Neurosci Lett, 2007. **429**(1): p. 1-6.

6. Mychasiuk, R., et al., *Dietary intake alters behavioral recovery and gene expression profiles in the brain of juvenile rats that have experienced a concussion.* Front Behav Neurosci, 2015. **9**: p. 17.

7. Haghighi, F., et al., *Neuronal DNA Methylation Profiling of Blast-Related Traumatic Brain Injury.* J Neurotrauma, 2015. **32**(16): p. 1200-9.

8. Wang, Y., et al., *Genome-wide screening of altered m6A-tagged transcript profiles in the hippocampus after traumatic brain injury in mice.* Epigenomics, 2019.

9. Lipponen, A., et al., *Transcription factors Tp73, Cebpd, Pax6, and Spi1 rather than DNA methylation regulate chronic transcriptomics changes after experimental traumatic brain injury.* Acta Neuropathol Commun, 2018. **6**(1): p. 17.

10. Gao, W.M., et al., *Immunohistochemical analysis of histone H3 acetylation and methylation--evidence for altered epigenetic signaling following traumatic brain injury in immature rats.* Brain Res, 2006. **1070**(1): p. 31-4.

11. Shein, N.A., et al., *Histone deacetylase inhibitor ITF2357 is neuroprotective, improves functional recovery, and induces glial apoptosis following experimental traumatic brain injury.* Faseb j, 2009. **23**(12): p. 4266-75.

12. Tai, Y.T., et al., *Low dose of valproate improves motor function after traumatic brain injury.* Biomed Res Int, 2014. **2014**: p. 980657.

13. Abu Hamdeh, S., et al., *Differential DNA Methylation of the Genes for Amyloid Precursor Protein, Tau, and Neurofilaments in Human Traumatic Brain Injury.* J Neurotrauma, 2021.

14. Israelsson, C., et al., *Distinct cellular patterns of upregulated chemokine expression supporting a prominent inflammatory role in traumatic brain injury.* J Neurotrauma, 2008. **25**(8): p. 959-74.

15. Samal, B.B., et al., *Acute Response of the Hippocampal Transcriptome Following Mild Traumatic Brain Injury After Controlled Cortical Impact in the Rat.* J Mol Neurosci, 2015. **57**(2): p. 282-303.

16. Zhang, X.Y., et al., *Analysis of key genes and modules during the courses of traumatic brain injury with microarray technology.* Genet Mol Res, 2014. **13**(4): p. 9220-8.

17. White, T.E., et al., *Gene expression patterns following unilateral traumatic brain injury reveals a local pro-inflammatory and remote anti-inflammatory response.* BMC Genomics, 2013. **14**: p. 282.

18. von Gertten, C., et al., *Genomic responses in rat cerebral cortex after traumatic brain injury.* BMC Neurosci, 2005. **6**: p. 69.

19. Hellmich, H.L., et al., *Pathway analysis reveals common pro-survival mechanisms of metyrapone and carbenoxolone after traumatic brain injury.* PLoS One, 2013. **8**(1): p. e53230.

20. Lamprecht, M.R., et al., *Strong Correlation of Genome-Wide Expression after Traumatic Brain Injury In Vitro and In Vivo Implicates a Role for SORLA.* J Neurotrauma, 2017. **34**(1): p. 97-108.

21. Lipponen, A., et al., *Analysis of Post-Traumatic Brain Injury Gene Expression Signature Reveals Tubulins, Nfe2l2, Nfkb, Cd44, and S100a4 as Treatment Targets.* Sci Rep, 2016. **6**: p. 31570.

22. Lipponen, A., et al., *In Vitro and In Vivo Pipeline for Validation of Disease-Modifying Effects of Systems Biology-Derived Network Treatments for Traumatic Brain Injury-Lessons Learned.* Int J Mol Sci, 2019. **20**(21).

23. Qin, X., et al., *Expression profile of plasma microRNAs and their roles in diagnosis of mild to severe traumatic brain injury.* PLoS One, 2018. **13**(9): p. e0204051.

24. Yang, T., et al., *Elevated serum miR-93, miR-191, and miR-499 are noninvasive biomarkers for the presence and progression of traumatic brain injury.* J Neurochem, 2016. **137**(1): p. 122-9.

25. Ge, X., et al., *MiR-21 alleviates secondary blood-brain barrier damage after traumatic brain injury in rats.* Brain Res, 2015. **1603**: p. 150-7.

26. Harrison, E.B., et al., *Traumatic brain injury increases levels of miR-21 in extracellular vesicles: implications for neuroinflammation.* FEBS Open Bio, 2016. **6**(8): p. 835-46.

27. Di Pietro, V., et al., *MicroRNAs as Novel Biomarkers for the Diagnosis and Prognosis of Mild and Severe Traumatic Brain Injury.* J Neurotrauma, 2017. **34**(11): p. 1948-1956.

28. Redell, J.B., et al., *Human traumatic brain injury alters plasma microRNA levels.* J Neurotrauma, 2010. **27**(12): p. 2147-56.

29. Balakathiresan, N., et al., *MicroRNA let-7i is a promising serum biomarker for blast-induced traumatic brain injury.* J Neurotrauma, 2012. **29**(7): p. 1379-87.

30. Sajja, V., et al., *Sphingolipids and microRNA Changes in Blood following Blast Traumatic Brain Injury: An Exploratory Study.* J Neurotrauma, 2018. **35**(2): p. 353-361.

31. Mitra, B., et al., *Plasma micro-RNA biomarkers for diagnosis and prognosis after traumatic brain injury: A pilot study.* J Clin Neurosci, 2017. **38**: p. 37-42.

32. Hicks, S.D., et al., *Overlapping MicroRNA Expression in Saliva and Cerebrospinal Fluid Accurately Identifies Pediatric Traumatic Brain Injury.* J Neurotrauma, 2018. **35**(1): p. 64-72.

33. Ko, J., et al., *Diagnosis of traumatic brain injury using miRNA signatures in nanomagnetically isolated brain-derived extracellular vesicles.* Lab Chip, 2018. **18**(23): p. 3617-3630.

34. Cheng, S.X., et al., *iTRAQ-Based Quantitative Proteomics Reveals the New Evidence Base for Traumatic Brain Injury Treated with Targeted Temperature Management.* Neurotherapeutics, 2018. **15**(1): p. 216-232.

35. Song, H., et al., *Quantitative Proteomic Study Reveals Up-Regulation of cAMP Signaling Pathway-Related Proteins in Mild Traumatic Brain Injury.* J Proteome Res, 2018. **17**(2): p. 858-869.

36. Thelin, E.P., et al., *Protein profiling in serum after traumatic brain injury in rats reveals potential injury markers.* Behav Brain Res, 2018. **340**: p. 71-80.

37. Xu, B., et al., *Protein profile changes in the frontotemporal lobes in human severe traumatic brain injury.* Brain Res, 2016. **1642**: p. 344-352.

38. Cherry, J.D., et al., *Characterization of Detergent Insoluble Proteome in Chronic Traumatic Encephalopathy.* J Neuropathol Exp Neurol, 2018. **77**(1): p. 40-49.

39. Abu Hamdeh, S., et al., *Proteomic differences between focal and diffuse traumatic brain injury in human brain tissue.* Sci Rep, 2018. **8**(1): p. 6807.

40. Huie, J.R., et al., *Testing a Multivariate Proteomic Panel for Traumatic Brain Injury Biomarker Discovery: A TRACK-TBI Pilot Study.* J Neurotrauma, 2019. **36**(1): p. 100-110.

41. Posti, J.P., et al., *Correlation of Blood Biomarkers and Biomarker Panels with Traumatic Findings on Computed Tomography after Traumatic Brain Injury.* J Neurotrauma, 2019. **36**(14): p. 2178-2189.

42. Ritzel, R.M., et al., *Chronic Alterations in Systemic Immune Function after Traumatic Brain Injury.* J Neurotrauma, 2018. **35**(13): p. 1419-1436.

43. Baker, E.W., et al., *Scaled traumatic brain injury results in unique metabolomic signatures between gray matter, white matter, and serum in a piglet model.* PLoS One, 2018. **13**(10): p. e0206481.

44. Jeter, C.B., et al., *Human traumatic brain injury alters circulating L-arginine and its metabolite levels: possible link to cerebral blood flow, extracellular matrix remodeling, and energy status.* J Neurotrauma, 2012. **29**(1): p. 119-27.

45. Jeter, C.B., et al., *Human mild traumatic brain injury decreases circulating branched-chain amino acids and their metabolite levels.* J Neurotrauma, 2013. **30**(8): p. 671-9.

46. Orešič, M., et al., *Human Serum Metabolites Associate With Severity and Patient Outcomes in Traumatic Brain Injury.* EBioMedicine, 2016. **12**: p. 118-126.

47. Fiandaca, M.S., et al., *Plasma metabolomic biomarkers accurately classify acute mild traumatic brain injury from controls.* PLoS One, 2018. **13**(4): p. e0195318.

48. Dickens, A.M., et al., *Serum Metabolites Associated with Computed Tomography Findings after Traumatic Brain Injury.* J Neurotrauma, 2018. **35**(22): p. 2673-2683.

49. Thomas, I., et al., *Integrative Analysis of Circulating Metabolite Profiles and Magnetic Resonance Imaging Metrics in Patients with Traumatic Brain Injury.* Int J Mol Sci, 2020. **21**(4).
